# Environmentally Friendly Approach to Pectin Extraction from Grapefruit Peel: Microwave-Assisted High-Pressure CO_2_/H_2_O

**DOI:** 10.3390/foods13030476

**Published:** 2024-02-02

**Authors:** Tuğba Öztürk, Hatice Neval Özbek, Derya Koçak Yanık

**Affiliations:** 1 Department of Food Engineering, Engineering Faculty, University of Gaziantep, Gaziantep 27310, Türkiye; ozturktuba24@gmail.com (T.Ö.); haticeneval@gantep.edu.tr (H.N.Ö.); 2 Department of Food Engineering, Faculty of Agriculture, Eskişehir Osmangazi University, Eskişehir 26160, Türkiye

**Keywords:** pectin, grapefruit peel, extraction, microwave-assisted high-pressure CO_2_/H_2_O

## Abstract

In this research, pectin extraction from grapefruit peel (GPP) was performed using a microwave-assisted high-pressure CO_2_/H_2_O (MW-HPCO_2_) system. The Box–Behnken design of response surface methodology was applied for the optimization of MW-HPCO_2_ extraction conditions to obtain the highest pectin yield. The effects of temperature, time, and liquid/solid ratio on pectin yield were examined in the range of 100–150 °C, 5–15 min, and 10–20 mL g^−1^, respectively. Under the optimum extraction conditions (147 °C, 3 min, and 10 mL g^−1^), pectin was obtained with a yield of 27.53%. The results obtained showed that the extraction temperature and time had a strong effect on the pectin yield, while the effect of the liquid/solid ratio was not significant, and the pectin was effectively extracted from grapefruit peel (GP) using MW-HPCO_2_. Additionally, the application of GPP in apricot jam showed that MW-HPCO_2_-GPP can be used as a thickener in the food industry. The yield and physicochemical properties (ash, protein, galacturonic acid, reducing sugar and methoxyl content, degree of esterification, equivalent weight, color, viscosity) of pectin extracted in the optimum conditions of the MW-HPCO_2_ method were superior to pectin extracted by the traditional method. The results of this study revealed that MW-HPCO_2_ could be an innovative green and rapid technique for pectin extraction.

## 1. Introduction

Citrus fruits (Rutaceae family) are fruits that are widely produced and consumed worldwide. Citrus fruits (orange, grapefruit, lemon, tangerine, etc.) contain large amounts of phytochemical and bioactive compounds as well as pectin [1]. Large amounts of citrus waste are generated in the food sector. This situation creates a negative burden for the environment. In citrus juice operations, approximately 50% of the whole fruit is waste, and 50–55% of this waste consists of peels. Pectin is found in nature in cell walls, intercellular spaces, and middle lamella of soil plants. Commercially, pectin is generally obtained from three main residues: sugar beet pulp, citrus peel, and apple pulp. Pectin-rich citrus peels (approximately 28% for oranges, 23% for grapefruit, and 19% for tangerines on a dry weight basis) are highly preferred in pectin production, and studies of pectin extracted from citrus peels have concluded that pectin contains rhamnogalacturonan I (RG I) and a small fraction of rhamnogalacturonan II (RG-II) [2,3,4]. Güzel and Akpınar [5] stated that the highest pectin yield was achieved from GP compared to other citrus peels. GP pectin is valuable due to its high RG-I content. GP pectin is widely preferred as a thickening, stabilizing, gelling, and emulsifying agent in varying industries. In terms of health, citrus pectin helps to regulate the gut microbiota, treat intestinal inflammation, and help the liver [5,6,7,8].

Pectin’s structure and amount are of critical importance in plants and vary depending on the region where the plants are located, the degree of ripening, and the storage and processing stages. The main components of pectic substances are D-galacturonic acid (GalA) and are attached at the α-1,4 position. The GalA content of pectin in citrus fruits varies between 58.5 and 85.4%. The linear structure of the poly-(GalA) chain of pectin is interrupted by β-1,2 linked L-rhamnose units, forming the RG-I region of pectin, which contains covalently linked sugars, the most important of which are D-galactose, L-arabinose, and L-rhamnose. The minor components of pectin are galacturonans such as rhamnogalacturonan-II and xylogalacturonan (XGA). RG-II consists of four different glycosidic complexes covalently cross-linked to at least eight units of the α-1,4 linked GalA backbone. These glycosidic complexes consist of several sugars. Xylogalacturonan is much less complex; its structure consists of a homogalacturonan substituted with xylose. Methyl groups esterify part of the carboxyl group at carbon 6 in the continuous poly-(GalA) chain; this indicates a change in the degree of methyl esterification (DE or DM); that is, DE gives the percentage of the ratio of esterified galacturonic acid units to the total galacturonic acid in pectin’s structure [9,10]. Pectin with a 50% and above DE is known as high-methoxyl pectin (HMP), while below 50%, it is known as low-methoxyl pectin (LMP). Generally, the esterification degree of commercial HMP is between 60 and 75%, while this rate is between 20 and 40% in LMP. The degree of esterification for various citrus fruits varies between 6.77 and 85.7%. These two different forms of pectin have different applications. For instance, HMP is able to form gels when heated in an acidic solution with a high sugar concentration (55–75%). On the other hand, LMP is able to form gels over an extensive pH range [9,11].

The extraction technique of pectin has a significant impact on pectin’s characteristics and yield. The conventional extraction (CE) method is based on the extraction of pectin with a hot diluted solution of strong mineral acids (such as sulfuric, nitric, phosphoric, acetic, or hydrochloric acid) followed by precipitation using ethanol. Some new technologies have been adopted in pectin extraction to overcome disadvantages such as long processing time, excessive energy consumption, the use of harmful chemicals, and the degradation of the pectin. Among these novel technologies, microwave and ultrasound energy, as well as subcritical water and enzymatic amplification of the extraction process, stand out [12]. In microwave-assisted extraction (MAE), microwave power provides rapid local heating and can lead to cellular rupture. In this way, pectin extraction becomes easier, the processing time is significantly shortened, and extraction yields are enhanced. Furthermore, microwave power can prevent pectinase enzyme activities that cause pectin degradation.

Microwave-assisted heating systems are considered a promising technique for processes such as extraction, synthesis, etc. However, the restricted penetration depth of microwave irradiation into absorbing materials is the main limitation of microwave scale-up processes. At present, this limitation is overcome by developing continuous or stop-flow microwave reactors. There are some studies in the literature that have demonstrated a successfully completed scale-up process of microwave technology in HMF production [13], polymerization [14], and flavonoid extraction [15].

Temperature or microwave power, time, and liquid/solid ratio are process parameters that have crucial impacts on the yield and quality of target compounds in microwave-assisted extraction. As the temperature increases, the diffusion rate of the solvent increases and the compound passes into the solvent, increasing the yield of the extract. However, prolonged processing at extreme temperatures can cause the degradation of heat-sensitive compounds, reducing the yield and/or purity of the target compounds [16]. In general, with increased extraction time comes increased yield. But sometimes, extending the time can reduce the yield and quality due to some undesirable reactions, such as enzymatic degradation, oxidation, burning, etc. The liquid/solid ratio also affects the extraction efficiency in MAE. Maran et al. [17] reported that pectin yield increased when increasing the liquid/solid ratio; however, it must be kept at an optimum level because if the solvent volume is too high, microwave energy may be insufficient to heat the solvent. Additionally, microwave radiations are nonionizing rays, do not cause changes on the chemical bonds of the compound, and therefore do not change the molecular structure of the compound. Hence, the compound in the cell can be obtained in high purity [18].

The use of water and CO_2_ as solvents is nontoxic and nonflammable, and safe for the sustainable environment. When CO_2_ dissolves in water, carbonic acid is produced and acidifies the water. This facilitates transfer of pectin to the solvent. Additionally, the polarity of water decreases at temperatures above the boiling point of water in a pressurized environment (such as 180 °C), subcritical conditions occur, and water acts as an organic solvent [16]. Many extractions have been combined and tested with microwave. For example, Tien et al. [19] obtained pectin from dragon fruit by microwave–ultrasound-assisted extraction. Xu et al. [20] obtained pectin from jackfruit peel by ultrasonic microwave-assisted extraction with citric acid. Lal et al. [21] combined microwave-assisted extraction with pulsed electric field for pectin extraction from jackfruit waste. 

Pectin is a unique ingredient with its versatile role and has benefits not only for the food industry, but also for the pharmaceutical and cosmetic industries. Pectin is currently produced by extraction methods that require harsh chemicals. There are limited numbers of studies on alternative environmentally friendly pectin extraction methods. Transformation of the traditional methods into safe methods is essential to achieve sustainable production systems. In this respect, the novelty of this study is that MW-HPCO_2_, an environmentally friendly method for pectin extraction, is discussed for the first time. The aim of this study was to extract pectin from grapefruit peel using a new environmentally friendly extraction method, MW-HPCO_2_ extraction. The aim was also to increase the pectin yield by keeping its quality characteristics. The effects of extraction time, temperature, and liquid/solid ratio on pectin yield were examined and optimized in the studied range by maximizing the yield using the Box–Behnken design of response surface methodology (RSM). Finally, we also aimed to investigate the gelation performance of GP pectin obtained by the new method in apricot jam.

## 2. Materials and Methods

### 2.1. Materials

Fresh grapefruits were supplied from a garden located in Kozan-Adana/Türkiye. The fresh grapefruits juices were removed with a citrus juicer. After this process, fresh GP was grated and cut into small pieces and stored in plastic sample bags at approximately +4 °C. Moisture content of fresh GP was analyzed by oven drying and was found to be 83 ± 0.152%. All chemicals used in this study were of analytical grade and purchased from Sigma Aldrich (St Louis, MO, USA). Apricots to be used for jam preparation were purchased from the local market in Gaziantep/Türkiye.

### 2.2. Grapefruit Peel Pectin Extraction

#### 2.2.1. Conventional Extraction

Conventional extraction was performed using the technique stated by Su et al. [22] and Wu et al. [23]. Fresh GP was weighed and distilled water was added at a liquid/solid ratio of 50 mL/g. The pH value was adjusted to 1.0 by using 0.1 M HCl. The mixture was heated for 180 min with continuous agitation in a 90 °C water bath. Then, the mixture was filtered using a filter paper to remove the GP residues from the pectin extract. After that, 2 times the volume of ethanol was added to the filtrate and this solution was kept at 4 °C for 16 h to precipitate the pectin. Finally, the pectin precipitate was recovered from the ethanol solution using filter paper. The remaining ethanol solution was centrifuged at 10,000 rpm for 10 min to recover residual pectin. The pectin remaining on the filter paper was washed twice with ethanol and left for at least 4 h at −70 °C prior to freeze-drying. The frozen pectin was dried by keeping it in a freeze-dryer for 3 days. Pectin yield was determined by the following Equation (1) [24]:

(1) $$Yield\left(\%,drybasis\right):\frac{weight\;of\;dried\;pectin(g)}{weight\;of\;grapefruit\;peel(g)}\times 100$$

#### 2.2.2. Microwave-Assisted High-Pressure CO_2_/H_2_O (MW-HPCO_2_) Extraction

MW-HPCO_2_ extraction was performed by using a microwave system (Milestone SynthWave, Sorisole, Italy) equipped with a CO_2_ gas inlet and a cooler. Fresh GP was weighed to obtain a liquid/solid ratio defined in the experimental design (Table 1) and added to a 1 L polytetrafluoroethylene vessel where the extraction would take place. Distilled water at 5 °C was added to ensure maximum CO_2_ dissolution. Total mass of the reaction vessel was kept constant (the total of peel and water was 176 g). The system was brought to a completely closed environment and CO_2_ gas was introduced. The microwave power was set to maximum power (1500 W) and the power varied between 0–1500 W during the process. Temperature (100, 125, and 150 °C), time (5, 10, and 15 min), and liquid/solid ratio (10, 15, and 20) were set to the values given in experimental design (Table 1). To ensure homogeneous distribution in the reaction vessel during the microwave processes, the speed of the magnetic stirrer present in the MW-HPCO_2_ system was set at 35%. When the extraction was completed, the temperature was reduced back to room temperature in a short time with the help of cooler in the system and then the system was opened to take out the reaction vessel from the system. Then, GP residues were removed by filtration from the reaction medium. Finally, pectin was precipitated using ethanol as described in the conventional extraction process.

### 2.3. Experimental Design and Optimization by Response Surface Methodology

The experimental design was created using Design Expert 7.1.6 (StatEase, Minneapolis, MN, USA) software. Three independent variables (temperature, time, and liquid/solid ratio) affecting extraction conditions and the effects of their interactions on pectin yield were examined by a three-factor-three-level Box–Behnken design of RSM. The limits of independent variables were 100–150 °C, 5–15 min, and 10–20 mL/g for temperature, time, and liquid/solid ratio, respectively (Table 1). After the experiments were performed at conditions given in the design, data analysis and optimization for the MW-HPCO_2_ extraction were also performed using the software.

Experimental data were fitted to a second-order polynomial model Equation (2) [25] and regression coefficients were obtained. Coefficient of determination R^2^ was used to evaluate the fitness of the model.

(2) $$Y=A_{0}+\sum_{i=1}A_{ii}X_{i}^{2}+\sum_{i=1}^{n}\sum_{j=i+1}^{n}A_{ij}X_{i}X_{j}$$

where Y is the response variable (pectin yield); A_0_, A_i_, A_ii_, and A_ij_ are the regression coefficients for intercept, linear, quadratic, and interaction terms, respectively; X_i_ and X_j_ represent the independent variables (i  ≠  j).

For optimization, independent variables were kept within the studied range and extraction conditions were optimized for maximum pectin yield (%).

### 2.4. Physicochemical Properties of the Extracted Pectin

#### 2.4.1. Moisture, Ash, and Protein Content

Moisture and ash content of pectin samples were analyzed by gravimetric method. For moisture content determination, samples were kept in an oven at 105 °C until constant weight. The ash content was determined after 6 h of ashing in an oven at 550 °C. The protein amount in GPP was obtained by Kjeldahl procedure, and 6.25 was used as the conversion factor. 

#### 2.4.2. GalA Content

GalA content was determined using the spectrophotometric method as reported by Rodsamran and Sothornvit [26] with minor modification. Briefly, 6 mL of sulfuric acid (98%) was added to 1 mL of pectin solution (20 mg/100 mL of deionized water), and the solution was mixed and left for 20 min. Afterwards, 200 µL of 0.1% carbazole solution (in ethanol) was added to the mixture and vortexed. It was left in a dark place for 2 h until a pink color appeared. Then, the absorbance of the solution was measured at 520 nm by a spectrophotometer (Optima SP 3000 Nano, Tokyo, Japan). GalA content was determined from a calibration curve constructed using a standard D-(+)-Galacturonic acid solution at different concentrations (0–200 µg/mL). Results were defined as mg of GA per g of pectin (mg/g). 

#### 2.4.3. Equivalent Weight and Methoxyl Content

According to the study of Rodsamran and Sothornvit [26], equivalent weight (Eq.W) and methoxyl content of pectin were determined. Briefly, distilled water at 25 °C was added to 0.5 g GPP and the volume was made up to 100 mL. It was stirred till completely dissolved (approximately 2 h). Then, 1 g of NaCl was added and the solution was titrated with 0.1 N NaOH using 5 drops of phenolphthalein indicator. Eq.W was determined by the following equation:

(3) $$Eq.W=\frac{pectin(mg)}{concentration\;of\;NaOH\left(N\right)\times volume\;of\;NaOH\left(mL\right)}$$

Methoxyl content of samples was analyzed using the neutralized solution prepared above. Briefly, 25 mL of 0.25 N NaOH was added to this solution and stirred at room temperature. Then, 25 mL of 0.25 N HCl was added and the solution was titrated with 0.1 N NaOH. The following equation was used for methoxyl content calculation:

(4) $$Methoxyl\;content\left(\%\right)=\frac{concentration\;of\;NaOH\left(N\right)\times volume\;of\;NaOH\left(mL\right)\times 3.1}{pectin(g)}$$

#### 2.4.4. Degree of Esterification 

The titration method given by Barış et al. [27] was applied to determine the degree of esterification of pectin samples. First, 250 mg of pectin sample was wetted with 2 mL of ethanol and dissolved in 50 mL distilled water at 40 °C for 2 h to ensure complete dissolution. The solution was then titrated with 0.1 N NaOH (V1) in the presence of five drops of phenolphthalein. After titration, 10 mL 0.1 NaOH was added and the solution was stirred for 15 min. Then 10 mL of 0.1 HCl was added for neutralization until the pink color disappeared completely. After that, this solution was titrated again with 0.1 N NaOH (V2) in the presence of 5 drops phenolphthalein till a pale pink color appeared. DE was determined by the following formula:

(5) $$DE\left(\%\right)=\frac{V2}{V1+V2}\times 100$$

#### 2.4.5. Reducing Sugar Content

The reducing sugar content of GPP was analyzed by the DNS method using the procedure given by Özbek et al. [28]. Briefly, 3,5- dinitrosalicylic acid (DNS) reagent was prepared using 1 g DNS, 30 g Rochelle salt, and 20 mL of 2 N NAOH. The prepared reagent was filtered and then diluted to 100 mL. Then 2 mL of DNS reagent was poured on 2 mL of pectin solution (5 mg/mL) and the mixture was heated at 90 °C for 10 min. Absorbance of the mixture was measured at 540 nm by a spectrophotometer (Optima SP 3000 Nano, Tokyo, Japan). Reducing sugar content was calculated from the calibration curve created using glucose at different concentrations (0.25–2 mg/mL).

#### 2.4.6. Color

The color of the dried pectin was analyzed by a colorimeter (Hunter Lab Color Flex, VA, USA) and the CIE color system (L*, a*, b*). The measured color parameters were expressed as L*: lightness (100 white and 0 black), a*: greenness/redness (+a* redness and −a* greenness) and b*: yellowness/blueness (+b* yellowness and −b* blueness). The color of the samples was measured at ambient temperature (approximately 22 °C) in three replicates.

#### 2.4.7. Viscosity

A solution (2% *w*/*v*) was prepared from extracted pectin samples by mixing at 80 °C for 20 min and their viscosities were measured at 25 °C using a rotationally programmable viscometer (DV3T extra, Brookfield Engineering Inc., Middleboro, MA, USA). RV06 was used as the spindle. 

#### 2.4.8. Fourier Transform Infrared Spectroscopy (FTIR) Analysis

The FTIR spectrum of pectin samples was determined at 4 cm^−1^ resolutions. Spectra were recorded with 15 scans at 4000–600 cm^−1^ wavelengths using an FTIR spectrometer (Perkin Elmer Spectrum 100 FTIR Infrared Spectrometer, PerkinElmer, Waltham, MA, USA).

### 2.5. Morphology 

The surface morphology of freeze-dried grapefruit peels (untreated GP, GP after MW-HPCO_2_ extraction and CE methods) was characterized using a Zeiss GeminiSem300 (Carl Zeiss Ltd. Co., Oberkochen, Germany) scanning electron microscope (SEM). Pectin samples were mounted on aluminum SEM stubs and coated with gold: palladium on an Emitech SC7620 (Kent, UK) spray coater. SEM images were taken at 5 kV and 4000× magnification.

### 2.6. Preparation of Apricot Jam with MW-HPCO_2_ Pectin

Apricot jam was prepared with minor modifications using the method of Nourmohammadi et al. [29]. Briefly, apricots were pureed with a blender. Then, 300 g of this puree was mixed with 0.5% MW-HPCO_2_-GPP and 10% sucrose. For the control sample, jam was prepared without pectin addition. In the control, an amount of apricot puree was kept constant and 10.5% sucrose was added. The mixture was taken into a stainless-steel pot and heated (85–95 °C). It was constantly stirred with a glass rod during the heating. The heater was turned off when the TSS reached 55–60° Brix.

### 2.7. Textural Properties of Jam

The firmness, consistency, and cohesiveness of the jam samples were evaluated using the TA.XT2i texture analyzer (Stable Micro System, Godalming, UK) with a 500 Newton load as was performed by Nourmohammadi et al. [29]. The same volume of jam samples was reserved for cyclic testing. The vessel diameter, height, and probe diameter were 49 mm, 70 mm, and 40 mm, respectively. The probe penetration rate was 10 mm/s and the depth was 25 mm. 

### 2.8. Statistical Analysis

All analyses were performed in triplicate. Results are given as mean ± standard deviation. The statistical significance of the data obtained for the comparison of pectin achieved by MW-HPCO_2_ and CE was analyzed with *t*-test at the 95% confidence interval using SPSS, version 22.0 (IBM, Crop., Armonk, NY, USA).

## 3. Results and Discussion

### 3.1. Modeling of Pectin Yield in MW-HPCO_2_ Extraction

The results of the experiments carried out at conditions defined in the Box–Behnken design were analyzed with multiple regression analysis to obtain a statistical model. The results best fitted to a second-order polynomial model. The backward elimination was applied to remove insignificant factors and interactions from the model. The model equation for pectin yield (Y1) is given below:

(6) $$Y1=21.95+9.97*A-1.40*B-0.8*C-2.71*A*B-1.6*A*C-1.7\;*B*C-7.55*A^{2}-1.65*B^{2}$$

The model predicted was significant at the 95% confidence level. High values of adjusted and predicted R^2^ and nonsignificant lack of fit indicated that the statistical model fit well with the data (Table 2). Adequate precision measures the signal to noise ratio and it is desirable that the ratio be greater than 4. This value was 22.6 and indicated that the model was convenient for navigating the design space. Using analyses of variance (ANOVA), the accuracy of the model and the linear and interactive effects of the factors on the pectin yield (Y1) were examined. ANOVA results showed that the linear effects of temperature and time were statistically significant (*p* < 0.05) on pectin yield, while the effect of liquid/solid ratio was insignificant (*p* > 0.05). Similarly, Bagherian et al. [30] reported that microwave power (temperature) and irradiation time were significantly effective on yield in pectin extraction from grapefruit albedo. As in MW-HPCO_2_ extraction, Whang et al. [31] also stated that the linear effect of solid: liquid ratio is insignificant for microwave-assisted pectin extraction from apple pomace. The effects of extraction temperature ∗ liquid/solid ratio (A × C) and time ∗ liquid/solid ratio (B × C) interactions were statistically nonsignificant (*p* > 0.05) on pectin yield, while temperature ∗ time (A × B) interaction was significant (*p* < 0.05).

### 3.2. Effect of MW-HPCO_2_ Extraction Parameters on Pectin Yield

The pectin yield obtained in MW-HPCO_2_ extraction from GP varied between 2.70% and 28% (Table 1). The lowest pectin yield was obtained as 2.7% at 100 °C for 2 min and a 15 mL/g liquid/solid ratio. The highest yield was obtained as 28% at extraction conditions of 150 °C, 5 min, and 10 mL/g. In MW-HPCO_2_, under these conditions, pectin yield was higher than previously reported pectin yield (20.93%) by Taşan and Akpınar [32], who performed the microwave-assisted pectin extraction with grapefruit peel. The yield obtained by the MW-HPCO_2_ system from GP was also higher than that reported by Cui et al. [6] for GP in acid (16.24% to 21.28%,) and alkali (17.93%, 24.52%) extractions. 

**Figure 1:**
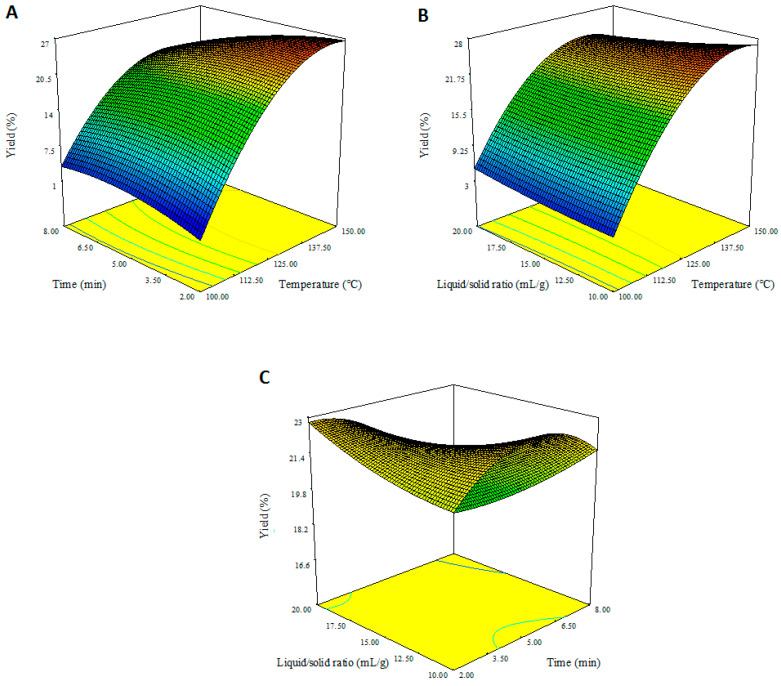
Effect of MW-HPCO_2_ extraction variables on pectin yield (%). (**A**) extraction temperature–time, (**B**) extraction temperature–liquid/solid ratio, (**C**) extraction time–liquid/solid ratio.

The 3D response surface plots clearly show relationships between dependent and independent factors. According to Figure 1A, the extracted pectin yield increased as the extraction time and temperature increased. It can be seen that pectin yield decreased at temperatures above 147 °C and with increasing hydrolysis time. Figure 1B shows the interaction of liquid/solid ratio and temperature. While the yield increased with increasing temperature, the liquid/solid ratio did not have a significant effect on the yield. Similarly, in Figure 1C, the yield increased with the increasing hydrolysis time; however, the liquid/solid ratio did not have a significant contribution to the yield.

### 3.3. Optimization and Validation of Model

The optimum extraction conditions estimated by RSM were 147 °C, 3 min, and 10 mL/g for temperature, time, and liquid/solid ratio, respectively. The desirability value for these conditions was 0.99. Pectin yield was estimated to be 27.74% under the predicted optimum conditions. Experimental validation was performed in triplicate and a pectin yield of 27.69 ± 0.07% was obtained. A one-sample *t*-test was applied to find significance between experimental and predicted yield. There was no significant difference (*p* > 0.05) between the predicted and experimental yield values, which means that the model was confirmed.

### 3.4. Comparison of MW-HPCO_2_ and CE Methods

Pectin yield varied depending on the extraction method. While the GPP yield obtained by MW-HPCO_2_ hydrolysis under optimum conditions was 27.69 ± 0.07%, the pectin yield obtained by CE method was 24.74 ± 0.07% (Table 3). MW-HPCO_2_ hydrolysis resulted in shorter processing time and higher pectin yield compared to CE method; this can be explained by combined effect of local heating and nonthermal effect (cell deformation) of microwave power, acidity caused by dissolving CO_2_, and high pressure during the process. Roman-Benn et al. [3] stated that grapefruit peels contain between 21.60% and 28.00% pectin. This suggests that MW-HPCO_2_ extraction is an effective method for extracting nearly all of the pectin from GP. Pectin yield achieved in this research was superior to the previously reported study by Taşan and Akpınar [32], who performed microwave pectin extraction from GP, as well as the reports provided by Cui et al. [6], who studied both acid and alkali extraction of pectin from GP. Unlike several MAE-based studies on pectin extraction from GP, in this study high-yield pectin was obtained without using any chemicals (acid or base). Furthermore, the extraction time was very short.

Moisture contents of MW-HPCO_2_-GPP and CE-GPP were determined as 4.52 ± 0.08% and 4.29 ± 0.03%, ash contents were determined as 2.88 ± 0.11% and 3.86 ± 0.15%, and protein contents were determined as 3.77 ± 0.10% and 3.67 ± 0.24%, respectively. The ash content of pectin is an important criterion for its purity and quality. Pectin with low ash content has higher purity and shows better gelling properties [5,33]. MW-HPCO_2_-GPP had a very low ash content compared to CE-GP because there is no use of any mineral acid in the MW-HPCO_2_ method. In a study performed by Chandel et al. [34], it was revealed that the acetyl groups and protein in the structure of pectin affect the emulsion-forming ability of pectin and increase emulsion stabilization. The protein content of pectin extracted from GP using MW-HPCO_2_ was higher than that of pectin extracted using acid and alkali, as reported by Cui et al. [6]. This suggests a superior emulsion property of the GPP obtained in this study. Compared to MW-HPCO_2_-GPP, the pectin derived from citrus limetta in a study performed by Sharma et al. [35] exhibited a high ash value and low protein value. Additionally, the protein content of pectin obtained from orange peel and lemon peel using the CE method was also low, as reported by El Fihry et al. [24]. Therefore, it can be inferred that MW-HPCO_2_-GPP has superior emulsification properties. 

GalA and DE values are used to determine pectin purity. The main constituent of the pectin backbone is GalA. The GalA contents of GPP obtained from MW-HPCO_2_ and CE were 83.90 ± 0.06% and 73.79 ± 0.23%, respectively (Table 3). The GalA content of MW-HPCO_2_ pectin was higher than that of CE pectin. Differences in GalA content may be related to the different pH applied in the methods. Higher acidity in CE may lead to lower GalA content [22].

The DE contents of MW-HPCO_2_-GPP and CE-GPP were 71.76 ± 0.81% and 69.30 ± 0.92%, respectively; the difference between them was insignificant (*p* < 0.05). Harsh conditions in CE (low-pH extraction and long process time) enhance the esterification of polygalacturonic chains, which may be the reason for the difference in GalA between them [22]. Similarly, in a study performed by Rodsamran and Sothornvit [26], microwave extraction of lime peel pectin had higher DE values than conventionally extracted pectin. DE is a significant factor for the gelation and emulsion capacity of pectin [35]. Liang et al. [36] stated that pea pectin with high DE showed greater interactions than pectin with lower DE, and that the emulsifying properties of citrus pectins at different DE also changed. DE values of GPP obtained by both methods are greater than 50% and can be classified as HMP. Additionally, it has the facility to form a fast-setting gel with DE > 70%. According to the GalA and DE results, the MW-HPCO_2_-GPP can be used commercially in food applications in which high methoxyl pectin is required.

The exact number of unesterified free galacturonic acid in the pectin chains is equal to the equivalent weight of pectin, and a high value of equivalent mass for pectin is a good indicator of gel formation [37,38]. MW-HPCO_2_-GPP had significantly higher (*p* < 0.05) equivalent weight compared to CE-GPP (Table 3). It may be due to less pectin degradation in the MW-HPCO_2_ method. Similarly, Rodsamran and Sothornvit [26] reported that equivalent weight of pectin achieved by microwave-assisted extraction was higher than that obtained by the traditional extraction method due to more pectin degradation in the traditional method.

The methoxyl percentage of pectin shows the number of methyl ester clusters on its structure and is an important factor determining its gel-forming ability, sensitivity to polyvalent cations, and setting time. It also represents the ability of pectin to disperse in water. The methoxyl percentage of MW-HPCO_2_-GPP was significantly higher (*p* < 0.05) than that of CE-GPP (Table 3). The methoxyl percentage of GPP obtained by both extraction techniques was higher than 7. This indicates that pectins obtained in this study are classified as high-methyl pectin (commercially 8–11% methoxyl content) [34,39]. Methoxyl contents greater than 7% indicate that pectin can form gels at high sugar concentration (>65% sugar). Therefore, the GPP obtained in the current study is easily dispersed in water, has high sugar-binding capacity, and may be suitable for the preparation of products such as jam and jelly, because products with high acidity and high sucrose concentration (>65%) require high methoxyl pectin [38].

Pectin monosaccharides are linked together by glycosidic bonds. The breakdown of these linkages leads to an increase in the reducing sugar content of pectin [40]. The reducing sugar contents of MW-HPCO_2_-GPP and CE-GPP were 7.68 ± 0.22% and 8.43 ± 0.23%, respectively. Reducing the sugar content is necessary for jam as it can increase the strength of the gel. Additionally, sucrose added to jam partially turns into invert sugar. High reducing sugar content of pectin may reduce its susceptibility to the Maillard reaction and may also indicate deterioration of the pectin structure [41,42]. 

The color of pectin is one of the important quality characteristics that determines its commercial value. Pectin color is significantly affected by its pigment content and phenolic compounds. Additionally, reducing the sugar content of pectin is another important parameter that affects its color through the Maillard reaction that occurs during the extraction process [35]. Pectin obtained with MW-HPCO_2_ had a lighter color (higher luminosity, L*) and higher yellowness (b*) values than that obtained by CE (*p* < 0.05), but the difference in redness (a*) value of GPP obtained by MW-HPCO_2_ and CE was not significant (*p* > 0.05). The dark color of CE-GPP can be explained by the long processing time that causes a browning reaction in CE.

The apparent viscosity of pectin is significantly related to its molecular weight. Both apparent viscosity and molecular weight of pectin obtained by MW-HPCO_2_ extraction were higher than those obtained by CE (Table 3). The difference in viscosity of GPP is associated with differences in extraction procedure, such as heating method, which results in different methoxyl content [26]. High molecular weight and high viscosity of pectin enhance emulsion stability [43]. Hence, MW-HPCO_2_-GPP can be useful as a thickener in foods or beverages. The viscosity of MW-HPCO_2_-GPP is considerably higher than the viscosity of citrus pectin and fig stalk pectin reported by Çavdaroğlu and Yemenicioğlu [44]. Also, Hu et al. [45] conducted a comparison of the viscosities of pectin extracted from waste citrus peel. Their results showed that grapefruit peel pectin exhibited a higher viscosity than other citrus pectins (lemon peel, orange peel, and pomelo peel pectin).

**Figure 2:**
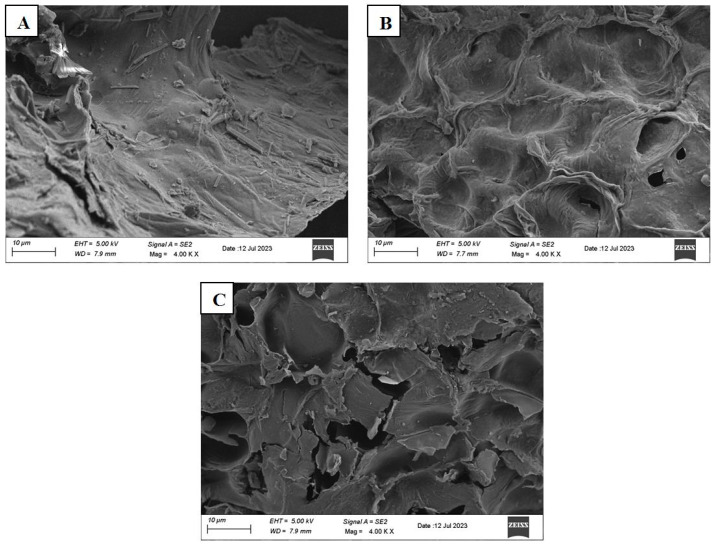
Scanning electron microscopy images of (**A**) untreated GP; (**B**) grapefruit peel after CE method; (**C**) grapefruit peel after MW-HPCO_2_ method.

SEM images of GP before and after pectin extractions are shown in Figure 2. It is clear to see that while untreated GP has a smooth flat surface structure, both CE and MW-HPCO_2_ extraction processes caused a dramatic change to GP by creating porous structure and pits on the GP surface. However, MW-HPCO_2_ extraction caused more deformation of the GP morphology with a wrinkled surface and numerous fragments. These results support the high extraction yield in MW-HPCO_2_. Previously, Lal et al. [21] studied the effect of microwave-assisted extraction combined with pulsed electric field on pectin extraction from jackfruit peels and reported that a major disintegration in the physical structure of jackfruit peel occurred after treatment, leading to an increase in the extraction yield. In this study, the greater fragmentation of GPs after MW-HPCO_2_ treatment compared to the CE method supports the significant differences in pectin yield between two methods. 

**Figure 3:**
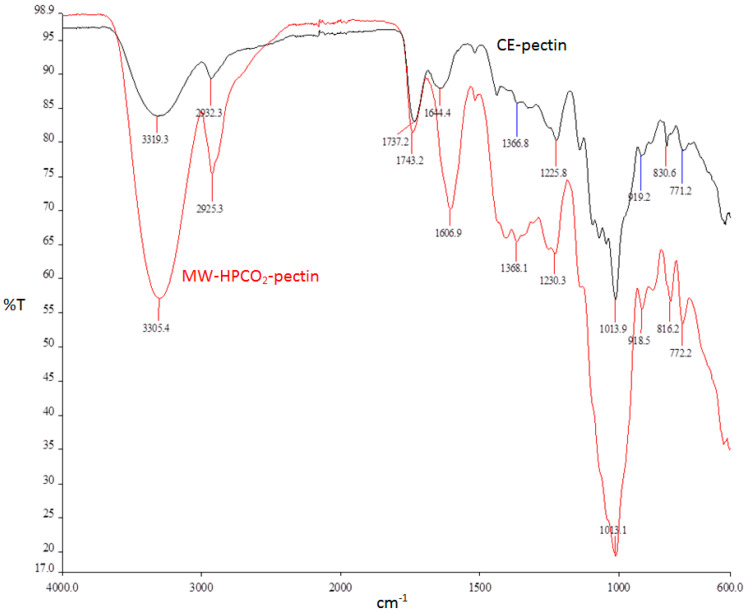
FTIR spectra of CE and MW-HPCO_2_ extracted GPP.

The FTIR spectra of MW-HPCO_2_-GPP and CE-GPP revealed that both pectins had same characteristic peaks and no difference was observed between them (Figure 3). Generally, the FTIR band of MW-HPCO_2_-GPP was more intense than that of CE-GPP. The samples extracted by MW-HPCO_2_ and CE techniques had typical absorption peaks of pectin, considering previous studies on pectin [32,43]. The large absorption peak at around 3300 cm^−1^ was caused by stretching of hydroxyl groups (O–H) and the peak at 2900 cm^−1^ was attributed to the C–H stretching of the CH_2_ groups. The absorption at 1743 cm^−1^ represents the C=O stretching vibration of ester carbonyl, while the absorption around 1600 cm^−1^ represents the C=O stretching vibration of the carboxyl group. The absorption peaks between 1000 and 1400 cm^−1^ are defined as the fingerprint region where C–H bending (pyranoid ring), C–O–C stretching (glycosidic bond), C–O stretching (alcohol), and CH_3_ bending (COOCH_3_) are located [46]. The absorption peaks at 1010 and 1150 cm^−1^ showed that the samples contain pyranose. The peaks at around 910 cm^−1^ and 816 cm^−1^ represent the D-glucopyranosyl and α-D-mannopyranose, respectively [47].

The texture properties (firmness, consistency, and cohesiveness) of apricot jam obtained with MW-HPCO_2_-GPP were evaluated and compared with the control sample (Table 4). The jam sample prepared with GPP had improved quality characteristics compared to the control sample. GPP made the apricot jam thicker, firm, and more cohesive. These results show that it is more difficult to break the gel system of MW-HPCO_2_-GPP apricot jam than the control sample and that the MW-HPCO_2_-GPP is suitable for use as an additive (thickener, gelling agent) for foods such as jam and jelly. Similarly, Bekele et al. [48] stated that the consistency of mango jam prepared without pectin was lower than that of the sample prepared using pectin.

## 4. Conclusions

The MW-HPCO_2_ method was successfully applied for pectin extraction from grapefruit peel. According to the Box–Behnken design of RSM, among the extraction parameters, temperature and extraction time had a significantly effect on pectin yield; however, liquid/solid ratio was not effective. Pectin was extracted rapidly and easily without any toxic chemicals or time-consuming extraction process. The MW-HPCO_2_ method is also capable of extracting more pectin than the conventional extraction method. Pectin extracted from GP using CE and MW-HPCO_2_ methods had different characteristics. Pectin obtained by the MW-HPCO_2_ method was better in terms of DE, GalA, Eq.W, methoxyl content, color, and apparent viscosity. Using grapefruit pectin obtained by the MW-HPCO_2_ method resulted in good textural characteristics in apricot jam. This result suggests that GP pectin may be an alternative to be used as a thickener in the jam industry. The results of this study also confirm that MW-HPCO_2_ is an environmentally friendly, efficient, and time-saving method compared to the traditional method. However, further studies should be performed to reveal the energy efficiency of the MW-HPCO_2_ system in the extraction process.

## Figures and Tables

**Table 1:** Box–Behnken design and experimental results for pectin extraction using MW-HPCO_2_.

Run Numbers	Factors			Response
	A: Temperature (°C)	B: Time (min)	C: Liquid/Solid Ratio (mL/g)	Pectin Yield (%)
1	150	2	15	27.00
2	100	2	15	2.70
3	125	5	15	20.90
4	125	8	10	22.00
5	100	5	10	3.80
6	150	5	10	28.00
7	125	2	10	19.60
8	100	5	20	4.40
9	125	5	15	23.00
10	125	5	15	22.66
11	150	8	15	17.00
12	150	5	20	22.20
13	125	5	15	20.50
14	125	8	20	18.00
15	125	5	15	21.90
16	100	8	15	3.53
17	125	2	20	22.40

**Table 2:** R^2^ values of model given by response surface methodology for dependent variables.

R^2^ Value	Response Pectin Yield (Y1)
R^2^	0.9860
Adjusted R^2^	0.9721
Predicted R^2^	0.8963
Adeq. Precision	24.686
Lack of fit	0.2086

**Table 3:** Physicochemical properties of extracted GPP samples.

Sample	CE-GPP	MW-HPCO2-GPP
Yield (%)	24.74 ± 0.07 ^a^	27.69 ± 0.07 ^b^
GalA (%)	73.79 ± 0.23 ^a^	83.90 ± 0.06 ^b^
DE (%)	69.30 ± 0.92 ^a^	71.76 ± 0.81 ^b^
Eq.W	2383.8 ± 2.19 ^a^	5049 ± 1.41 ^b^
Methoxyl content (%)	8.93 ± 0.05 ^a^	10.44 ± 0.08 ^b^
Reducing sugar (%)	8.43 ± 0.23 ^a^	7.68 ± 0.22 ^b^
Color		
L*	74.69 ± 0.35 ^a^	90.68 ± 0.22 ^b^
a*	1.43 ± 0.09 ^a^	1.42 ± 0.11 ^a^
b*	23.36 ± 0.49 ^a^	21.10 ± 0.10 ^b^
Viscosity (mPa.s)	16.67 ± 0.43 ^a^	31.37 ± 0.63 ^b^

CE-GPP: grapefruit peel pectin obtained by conventional extraction; MW-HPCO_2_-GPP: grapefruit peel pectin obtained by MW-HPCO_2_; GalA: galacturonic acid; DE: degree of esterification; Eq.W: equivalent weight. Data are presented as mean ± standard deviation. Different superscript letters in the same row differ significantly (*p* < 0.05).

**Table 4:** Results of texture properties of apricot jam samples.

Apricot Jam Samples	Firmness	Consistency	Cohesiveness
Control	398.72 ± 2.54 ^a^	883.30 ± 4.77 ^a^	321.51 ± 0.78 ^a^
MW-HPCO_2_-GPP	459.47 ± 8.37 ^b^	1009.29 ± 13.13 ^b^	393.23 ± 1.44 ^b^

Control: jam without pectin; MW-HPCO_2_-GPP: jam with MW-HPCO_2_-GPP. Data are presented as mean ± standard deviation. Different superscript letters in the same column differ significantly (*p* < 0.05).

## Data Availability

The original contributions presented in the study are included in the article, further inquiries can be directed to the corresponding author.
